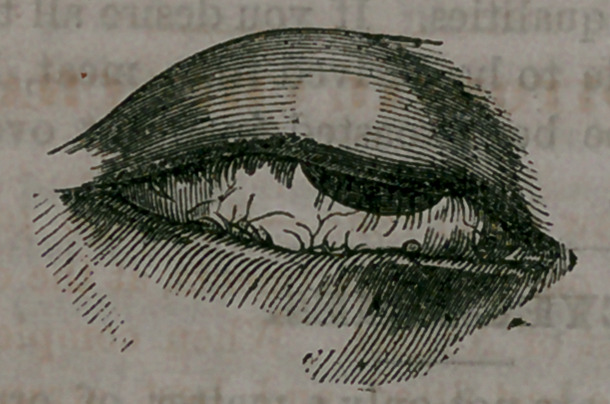# Eyelashes

**Published:** 1871-07

**Authors:** 


### EYELASHES.

The eyelashes are not only a matter of ornamentation to the eyes, adding to their grace and beauty, but are also highly useful in preventing particles of foreign substances reaching the eye, such as dust, sand, cinders, &c. But, it sometimes happens that the lashes become a great source of irritation to the eyes, producing serious inflammation, and not infrequently destroying vision. This happens when they are irregular in their growth, when, instead of arching gracefully outward from the eye, they become straggly, pale, thin and stunted in growth, and turn in upon the globe. Sometimes they are so pale and delicate as not to be discovered by the naked eye, still, the patient is aware of their presence by reason of the constant irritation produced, causing a sensation as of sticks or sand in the eye. Often, but two or three delicate cilica are involved in the malposition, but, through the constant irritation excited, finally involve the entire row. Frequently a double row of lashes are seen, the outer one being in a natural position, while the inner, composed of very fine and delicate hairs, lay directly upon the eye. The affection is common to both the upper and lower lids, and is equally dangerous in either position. The usual causes for such deformities are the plucking out of the eye-lashes for the removal of supposed "wild hairs;" severe applications to the under surface of the eye-lids, for the removal of granulations; inflammation of the margins of the lids, which destroy or so impair the functions of the bulbs of the hair as to distort their growth; styes, abscesses, or wounds at the edge of the lids, by which the bulbs are injured, are fruitful causes for the difficulty.

When, from the thickening of the edge of the lid, the lashes have arranged themselves into two rows, the inner of which lies upon the globe, the ophthalmic surgeon names the disease *districhiasis.* If the malposition extends to the cilia generally, it is then named *trichiasis*, and presents an appearance as represented in the following illustration, where the straggling and inturning condition of the lashes, with the thickened and distorted margins of the lids, are accurately illustrated:

The application of medicines, with a view to correcting such a difficulty as this, is worse than useless. Salves, poultices, and patent eye-waters, are powerless to correct such a deformity, and only tend to make matters worse. The continued plucking out of the lashes, weekly, by the patient or his medical attendant, only gives temporary relief, and eventually causes so much irritability of the lid as to cause *all* the lashes to turn upon the eye. Relief can only be had of a skillful ophthalmic surgeon, who has several means of cure at his hand. If the offending cilia be few, he will cut down to their bulbs and destroy them by means of a suitable cautery; or will pass a delicate silk thread through the bulbs, so destroying them, and preventing their growth. If the entire margin of the lid is involved, he will perform an operation by means of which the cilia are transplanted near their natural position, and thus instantly and completely arrest the disease, giving the patient immediate relief from his suffering.

Another very prevalent form of inturning lashes is found in cases where the lid itself turns inward, throwing the entire row of lashes upon the eyeball. This differs from *trichiasis* in that the lashes themselves are not at fault, they being perfectly even and natural, not being in the least irregular, nor stunted in growth, but are rolled in upon the eye-ball, by means of the inverted condition of the free edge of the lid. This form of the difficulty is called *entropium*, and is usually the result of bandaging the eye, in cases of severe inflammation, in order to protect it from light. The constant application of the bandage throws the lashes upon the ball, and are kept there by the spasmodic contraction of the lid in attempting to exclude the light. In such cases a superabundance of skin will be found laying in folds upon the lid, which can be taken up between the thumb and finger and the lashes so rolled off from the eyeball. In the more chronic form of of this disease, or where it is the result of long continued granulations of the lids, which have been the seat of severe inflammation, from irritable applications or cauterizations to their surfaces, the lid is contracted upon its under surface, and in this manner causes the inversion of the lashes. Long continued *trichiasis* will also, eventually, result in *entropium* or complete inversion of the lashes.

There is no disease of the eye more distressing to the patient or more disastrous in its results, than *entropium.* The lashes act as a constant irritant to the eye ball,producing great inflammation and ulceration of the cornea, and finally, entire destruction of the eye. In this condition the eye is very sensitive to the light, so that the patient is either compelled to sit in a dark room or to have the eyes closely bandaged, which only tends to press the lashes more firmly upon eye and so hasten destruction. We never see a distressing case of this character, without lamenting that the patient should not know that relief is so easily afforded. They generally submit to the home treatment of every old granny, who invariably is in possession of a cure, which is tried and plied until vision is completely ruined. This negligence or ignorance is all the more deplorable when it is known that an ophthalmic surgeon can afford *instant* and permanent relief, by means of a very simple operation, which can be completed in less than one minute: A sufficient fold of the relaxed skin of the lid is taken up,with a suitable instrument,until the lid is caused to roll out sufficiently to clear the lashes from the eyeball. This superfluous skin is then snipped out with the scissors, and the margins of the wound brought together by means of delicate threads. This procedure at once turns the lashes from the eye and prevents their inturning, so that relief is immediately afforded. The wound heals perfectly within three days, and the cure is complete. If much injury is done to the eye from the irritation produced by the lashes,—and this is generally the case—then treatment can be adopted with a view of restoring vision, but, until after the *cause* of such injury has been removed, all treatment, in the way of local applications, will be found to be worse than useless.

Traveling doctors and patent medicines are not the means that should be sought, in this or any other difficulty of the eyes, for a cure.—At this day, skillful specialists can be found in almost every city of the Union; by applying to them in season you can not only relieve yourself of a most distressing disease of the eyes, but preserve your vision as well.

## Figures and Tables

**Figure f1:**